# Are there differences in birth weight between neighbourhoods in a Nordic welfare state?

**DOI:** 10.1186/1471-2458-7-267

**Published:** 2007-09-26

**Authors:** Eva Sellström, Göran Arnoldsson, Sven Bremberg, Anders Hjern

**Affiliations:** 1 Department of Health Sciences, MidSweden University, S-831 25 Östersund, Sweden; 2 Department of Statistics, Umeå University, Umeå, Sweden; 3 Department of Public Health Sciences, Karolinska Institute, Stockholm, Sweden; 4 National Public Health Institute, Stockholm, Sweden; 5 Centre for Epidemiology, Swedish National Board on Health and Welfare, Stockholm, Sweden; 6 Department of Children's and Women's Health, Uppsala University, Uppsala, Sweden

## Abstract

**Background:**

The objective of this cohort study was to examine the effect on birth weight of living in a disadvantaged neighbourhood in a Nordic welfare state. Birth weight is a health indicator known to be sensitive to political and welfare state conditions. No former studies on urban neighbourhood differences regarding mean birth weight have been carried out in a Nordic country.

**Methods:**

A register based on individual data on children's birth weight and maternal risk factors was used. A neighbourhood characteristic, i.e. an aggregated measure on income was also included. Connections between individual- and neighbourhood-level determinants and the outcome were analysed using multi-level regression technique. The study covered six hundred and ninety-six neighbourhoods in the three major cities of Sweden, Stockholm, Göteborg and Malmö, during 1992–2001. The majority of neighbourhoods had a population of 4 000–10 000 inhabitants. An average of 500 births per neighbourhood were analysed in this study.

**Results:**

Differences in mean birth weight in Swedish urban neighbourhoods were minor. However, gestational length, parity and maternal smoking acted as modifiers of the neighbourhood effects. Most of the observed variation in mean birth weight was explained by individual risk factors.

**Conclusion:**

Welfare institutions and benefits in Sweden might buffer against negative infant outcomes due to adverse structural organisation of urban neighbourhoods.

## Background

Birth weight is an important indicator of infant health. Several individual risk factors like age, gestational age, preterm births, multiple pregnancies and diseases such as pre-eclampsia influence birth weight [1]. Previous research has also revealed a consistent social gradient in infant birth weight [2]. Race, maternal education, income and marital status are associated with the birth weight of a child [3-5]. This variation might partly be explained by the pregnant woman's exposure to stress [6,7]. Another explanation is differences in women's health related behaviours like smoking during pregnancy [8,9]. However, this research has an individual focus and cannot fully explain the variation in infant birth weight.

Accordingly, recent research has turned to contextual factors to provide a more complete explanation of the sources of social disparities in birth weigh [10,11]. Living in disadvantaged neighbourhoods could increase vulnerability to stress, influence maternal health status and health behaviours and thus increase the risk of giving birth to a low weight infant. Women in disadvantaged US neighbourhoods were shown to have a 10–20% increased risk of giving birth to an infant with low weight or intra-uterine growth retardation [12-19]. Similarly, in a study by Spencer et al 10 percent of the variation in mean birth weight was explained by area deprivation level [20]. Similar neighbourhood effects have been demonstrated in other British studies [21,22].

We found no previous studies on urban neighbourhood differences regarding mean birth weight from a Nordic country. Yet, there is a pattern of segregation also in Swedish neighbourhoods which has been comprehensively described by geographers such as Andersson and Molina [23,24]. Swedish registers report that several indicators of individual vulnerability, such as unemployment, low income, receipt of social allowance, poor school achievement, bad health and exposure to violence, are closely linked to the income structure of the neighbourhood [25,26]. Moreover, in a recent report it was clarified that the ethnic segregation has increased and coincide progressively more with economic segregation [27].

Put together, the evidence of an impact of residential segregation on birth weight is based on research stemming from liberal Anglo-Saxon nations where the accent is on a minimal state and "as much market as possible" [28]. Furthermore, birth weight is a health indicator known to be sensitive to political and welfare state conditions [29-33]. Chung and Muntaner could confirm an association between welfare state health policies and child health outcomes [34,35]. Characteristics of the Nordic model of welfare state include a broad scope of social policies, universal social benefits, free or subsidised services, a high proportion of social expenditure of GNP, and a relatively even income distribution [28].

In this study we aim to investigate any neighbourhood effects on birth weight in the Swedish society. As demonstrated above, neighbourhoods in the major cities of Sweden exhibit rather great social differences. However, negative health effects of living in disadvantaged neighbourhoods might be modified by a system of equalising resources. The following three specific research questions were addressed in the study:

1. What is the effect on birth weight of living in a disadvantaged neighbourhood, when individual-level factors, i.e. mothers' social position and other relevant confounders, are controlled for?

2. How much of the variation in birth weight between areas can be attributed to the neighbourhood composition of individuals [i.e. intra-class correlation]?

3. How much of the residual variation in birth weight between neighbourhoods can be explained by neighbourhood economic status after controlling for individual determinants?

## Methods

### Study population

The study population consisted of 330 096 singleton live births to mothers who delivered between 1992 and 2001 in the three major cities in Sweden, Stockholm, Göteborg and Malmö. The cities were divided into 696 neighbourhoods. Neighbourhoods with < 500 inhabitants or in which no infants were born during a study year were excluded from the analyses (Table 1). Of the 330 096 singleton births 1 717 (0.5%) occurred in neighbourhoods with < 500 inhabitants. In addition, 502 births (0.2%) could not be mapped to a neighbourhood. Thus, 327877 births were analyzed. Characteristics of excluded cases are similar to those included with respect to education, smoking habits, age and parity. Depending on the year of the study, between 104 and 115 neighbourhoods were excluded. The majority of neighbourhoods had a population of 4 000–10 000 inhabitants. On average 500 births per neighbourhood were analysed in this study. Table 1.

**Table 1:** Classification of and descriptive statistics on neighbourhood economic status

**Neighbourhood economic status**	**Code**	**LH-ratio**	**Births (n)**	**%**	**N-hood economic status (recoded)**	**Code**
Very high level of resources	1	< 0.25	6 105	1.8	Affluent	1
High level of resources	2	0.25–0.49	37 135	11.2		
Medium to high level of resources	3	0.50–0.79	64 337	19.5	Medium level	2
Medium level of resources	4	0.80–1.24	73 969	22.4		
Medium to low level of resources	5	1.25–1.99	59 678	18.1		
Low level of resources	6	2.00–3.99	45 288	13.7		
Very low level of resources	7	4.00–9.99	27 084	8.2	Poor	3
Poor	8	> 9.99	14 281	4.3		

**Total**			**327 901**	**99.3**		

Missing (< 500 inhabitants)			1693	0.5		
Missing (no information on neighbourhood)			502	0.2		

Total			330 096	100.0		

### Design and variables

This study was based on the Social Database in which Swedish national registers held by The Swedish National Board of Health and Welfare and Statistics Sweden are linked through each individual's unique civic registration number [36-39]. Data on pregnancy and on birth weight of the infant were derived from the Swedish Medical Birth Register held by the Swedish National Board of Health and Welfare [37]. Information on the pregnant woman's socio-economic status were taken from the LOUISE database of Statistics Sweden [38,39]. Neighbourhood economic status, i.e. an aggregated measure on income, was also used. Individual and ecological data were linked via a geographical code for each neighbourhood [39,40]. Data were analysed using multi-level linear regression [41,42].

### The outcome variable

In this study we have used mean birth weight as outcome variable. Birth weight is an outcome with good validity and high coverage [43]. Many studies rely on a dichotomous measure of low birth weight, which is defined as a birth weight less than 2500 g. This is a convention that dates back to 1919 [1]. More recent research has shown that low birth weight is a threat to infant health especially if it is a result of intrauterine growth retardation. Furthermore, perinatal and infant mortality is lowest in births > = 3500 g [44]. Besides, low birth weight is a rare outcome which makes it more difficult to detect any neighbourhood variation. In the analysis, we controlled for gestational age. Thus, to use a linear specification of the outcome has a greater possibility to capture potentially critical variation across the distribution of birth weight that cannot be explained by length of gestation.

### Individual-level predictors

Data on mothers' height, age, length of gestation, parity and smoking habits during pregnancy (coded as 0 = 0 cigarettes daily; 1 = one to nine cigarettes daily; 2 = ten or more cigarettes daily) were derived from the Medical Birth Register [37]. Absurd values on maternal height were removed before the analyses. For the purpose of the multi-level analysis, maternal height was centred around 165 cm. Data on mothers' educational backgrounds were obtained from the Swedish Education Register, and coded so that 4 = university education; 3 = 3–4 years of secondary school; 2 = 2 years of secondary school; and 1 = primary school [38]. Data on educational background were identified 2 years before the year of study.

### The neighbourhood-level

Areas in the region are defined as neighbourhoods based on natural geographic borders and homogeneity of housing [45]. The aggregation of neighbourhoods is based on a system of geocoding in which all estates in Sweden are allocated a key code [40,45]. The social database contains an aggregated measure on each neighbourhood's economic status [45]. The variable is updated every year and for the purpose of this study neighbourhood data were collected on 31 December of the year before the birth of each infant included in the cohort. For each neighbourhood, one observation was made per study year, giving ten independent observations per neighbourhood.

'Economic status' was calculated as the ratio of low-to high-income earners in the neighbourhood (LH-ratio). Income earners are divided into three classes, low, normal and high. Low income earner is defined as belonging to the lowest quintile of income earners in the actual region; Stockholm, Malmö, Göteborg. In 1990 persons with an income below 123,300 SEK, 114,500 SEK and 123,800 SEK respectively were defined as low income earners. In 2002 corresponding limits were 151,600 SEK, 121,600 SEK, 150,200 SEK. High income earner is defined as belonging to the highest quintile of income earners in the region. In 1990 persons with an income above 255,400 SEK, 225,300 SEK and 232,500 SEK respectively were defined as high income earners. In 2002 corresponding limits were 406,800 SEK, 352,400 SEK, 364,600 SEK. Thus, in affluent neighbourhoods, where high income earners are more numerous than low income earners, this ratio is far less than 1. Correspondingly, in poor areas with more low income earners the ratio by far exceeds 1.

The rationale for using this ratio is that not only the prevalence of low income earners in an area but also of high income earners plays an important role for the neighbourhood social climate. Having a high income coincides for example with better education and high income household can be assumed to have a strong demand on public and commercial services of good quality, such as schools, primary care etc in their neighbourhood. Accordingly, the presence of high income households constitutes a stabilizing factor within the neighbourhood and as such is beneficial to the whole area.

Table 1 illustrates how the neighbourhoods are classified and how births are distributed over neighbourhoods. For the purpose of bivariate analyses, neighbourhood economic status was recoded into three categories.

### Analytic approach

The multilevel analyses were conducted using hierarchical modelling technique [41,42]. We estimated six models for birth weight as a continuous outcome variable. Model 1 was the simplest model, the unconditional model with no predictor variables specified. Birth weight was free to vary across two levels of analysis: individual (level 1) and neighbourhood (level 2). From the unconditional model the degree of resemblance between infants belonging to the same neighbourhood, the intra-class correlation (ICC), was calculated. Intra-class correlation coefficient is the proportion of variance that is accounted for by the neighbourhood level, according to the following formula:

P=σu2σu2+σe2∗100,
 MathType@MTEF@5@5@+=feaafiart1ev1aaatCvAUfKttLearuWrP9MDH5MBPbIqV92AaeXatLxBI9gBamXvP5wqSXMqHnxAJn0BKvguHDwzZbqegyvzYrwyUfgarqqtubsr4rNCHbGeaGqiA8vkIkVAFgIELiFeLkFeLk=iY=Hhbbf9v8qqaqFr0xc9pk0xbba9q8WqFfeaY=biLkVcLq=JHqVepeea0=as0db9vqpepesP0xe9Fve9Fve9GapdbaqaaeGacaGaaiaabeqaamqadiabaaGcbaGaemiuaaLaeyypa0ZaaSaaaeaacqaHdpWCdaqhaaWcbaGaeeyDauhabaGaeGOmaidaaaGcbaGaeq4Wdm3aa0baaSqaaiabbwha1bqaaiabikdaYaaakiabgUcaRiabeo8aZnaaDaaaleaacqqGLbqzaeaacqaIYaGmaaaaaOGaey4fIOIaeGymaeJaeGimaaJaeGimaaJaeiilaWcaaa@519E@

where σu2
 MathType@MTEF@5@5@+=feaafiart1ev1aaatCvAUfKttLearuWrP9MDH5MBPbIqV92AaeXatLxBI9gBaebbnrfifHhDYfgasaacH8akY=wiFfYdH8Gipec8Eeeu0xXdbba9frFj0=OqFfea0dXdd9vqai=hGuQ8kuc9pgc9s8qqaq=dirpe0xb9q8qiLsFr0=vr0=vr0dc8meaabaqaciaacaGaaeqabaqabeGadaaakeaacqaHdpWCdaqhaaWcbaGaeeyDauhabaGaeGOmaidaaaaa@30FF@ denotes the neighbourhood-level variance and σ_e_^2 ^is the variance at the individual level.

Thereafter we elaborated the random intercept and random slope models, consisting of two equations estimated simultaneously, an individual-level model and a neighbourhood-level model [42]. The variables included one observation for each year. Therefore we calculated the neighbourhood variance as a function of year of birth:

Y_ij _= β_0j _+ β_1j _+ β_1j_(year of birth - 1996) + β_1 _X_1ij _+ ... + β_p _X_pij_+ e_ij_,

where *Y*_*ij *_is the infant's birth weight for mother *i *in neighbourhood *j; β*_0*j *_is the overall constant (intercept); *β*_1*j *_is the random slope for year of birth in neighbourhood *j*, *β*_1_*X*_1*ij *_+...+ *β*_*p *_*X*_*pij *_are the partial effects of individual-level variables on birth weight; and *e*_*ij *_is the individual-level variation in birth weight.

In the neighbourhood-level model, model 3, the intercept from the individual-level, *β*_0*j*_, is allowed to vary randomly across neighbourhoods, as follows:

β_0j _= *γ*_00 _+ ***γ***_1j _+ ***γ***_01_Z_1j _+...+ *γ*_0q_Z_qj _+ u_0j_,

where *γ*_00 _is the average-value birth weight across all neighbourhoods, ***γ***_1j _is the average slope across the years of birth included in the model, and *γ*_0q _is the neighbourhood-level effect; Z_qj _is a neighbourhood-level determinant; and u_0j _is the variation at the neighbourhood level. The neighbourhood-level model estimated neighbourhood effects before adjusting for individual-level covariates.

Models 4–6 added individual maternal risk factors. Biological factors, socio-demographic variables and maternal smoking were entered in three steps. Non-significant variables were excluded from further analyses.

Finally, relevant cross-level interactions between significant neighbourhood and individual-level variables were explored. No interaction variables reached significance and were therefore not entered in the model.

Variance components for the individual- and neighbourhood-level models are presented. The between-neighbourhood variation was described as the proportional change in variance in the individual-level model and the neighbourhood-level model compared with the null model [42]. Parameters were estimated using iterative generalized least squares (IGLS). We also ran the models using MCMC procedures. The results were similar thus indicating robustness of estimates. WALD statistics were used to assess significance of estimates and a value of > 3 was considered significant. The MLwiN version 2.01 software was used to perform the multi-level modelling [46].

## Results

Table 2 shows how infants born in poor neighbourhoods had 106 g lower weight, on average, than did infants from affluent neighbourhoods and 31 g lower weight than did infants from medium economic status neighbourhoods (Table 2). The difference in mean birth weight was significant (one-way analysis of variance (ANOVA): F = 734.8, p < 0.001). In poor neighbourhoods a larger proportion of pregnant women were smokers, young (< 24 years old), multi-parous, and had low education and lower mean height compared to women from affluent and medium economic status neighbourhoods. All observed differences were highly significant.

**Table 2:** Descriptive characteristics of mothers and their newborn infants byneighbourhood economic status (N = 327901)

**Variable**	**n per variable (% of total)**	**Affluent neighbourhoods**	**Medium neighbourhoods**	**Poor neighbourhoods**	**Test of significance (ANOVA or CHI-square)**
Birthweight (g)	326469	3603	3572	3497	F = 734.8, df = 2,
Mean (SD)	(99.6%)	(536)	(549)	(556)	p < 0.001
Maternal height (cm)	291555	167.8	167.0	164.8	F = 3954.8, df = 2,
Mean (SD)	(88.9%)	(6.1)	(6.1)	(6.6)	p < 0.001
Gestational length	327402				Chi-value = 90.8,
22–28 weeks	(99.8%)	0.2%	0.2%	0.2%	df = 8
29–32 weeks		0.5%	0.6%	0.7%	P < 0.001
33–36 weeks		3.4%	3.8%	4.1%	
37–41 weeks		95.1%	94.4%	93.9%	
≥ 42 weeks		0.8%	1.0%	1.1%	
Parity	327901				Chi-value= 4354.0,
1	(100%)	38.5%	45.5%	40.7%	df = 4
2–3		57.7%	50.2%	49.6%	P < 0.001
≥ 4		3.8%	4.4%	9.7%	
Maternal age	327901				Chi-value= 15673.7,
12–19 yrs	(100%)	0.3%	1.0%	2.8%	df = 10
20–24 yrs		4.4%	11.6%	22.0%	P < 0.001
25–29 yrs		26.2%	35.1%	35.1%	
30–34 yrs		43.7%	35.2%	26.4%	
35–39 yrs		21.4%	14.5%	11.4%	
≥ 40 yrs		4.0%	2.7%	2.3%	
Smoking habits	301715				Chi-value= 3637.5,
0 cig/day	(92.0%)	92.7%	85.6%	79.8%	df = 4
1–9 cig/day		5.2%	9.4%	12.3%	P < 0.001
≥ 10 cig/day		2.1%	5.0%	7.9%	
Maternal education	321230				Chi-value= 30688.3,
Elementary	(98.0%)	5.1%	11.1%	27.7%	df = 8
Secondary (2 yrs)		36.5%	49.6%	50.6%	P < 0.001
Secondary (3–4 yrs)		23.8%	20.0%	12.3%	
University		34.6%	19.3%	9.4%	

SD = standard deviation.

The results of the multi-level analysis for continuous birth weight are presented in Table 3. In model 1, the null model, the observed ICC indicates a very small variation in birth weight between neighbourhoods.

**Table 3:** Multi-level multiple regression analysis of determinants of birth weight (as a continuous outcome) (n = 327877)

	**Model 1**	**Model 2**	**Model 3**	**Model 4**	**Model 5**	**Model 6**
Intercept	3562.58	3563.328	3624.578 (10.368)	3511.860 (8.732)	3518.116 (8.786)	3519.353 (8.713)
	(2.373)	(2.384)				
Year of birth		3.653 (0.363)	3.943 (0.365)	4.076 (0.335)	3.928 (0.336)	1.867 (0.337)

***Fixed effects***	***Beta (SE)***					

Neighbourhood economic status						
Very high level of resources			-	-	-	-
High level of resources			-27.806 (10.938)	-7.206 (9.202)	-5.380 (9.064)	-4.193 (9.180)
Medium to high level of resources			-36.764 (10.850) *	-0.982 (9.024)	2.372 (8.890)	6.625 (9.002)
Medium level of resources			-48.418 (10.776) *	-2.295 (8.961)	2.573 (8.837)	10.105 (8.942)
Medium to low level of resources			-66.231 (10.845) *	-8.818 (9.025)	-2.862 (8.908)	8.061 (9.006)
Low level of resources			-91.652 (11.000) *	-23.591 (9.148)	-14.204 (9.049)	-1.210 (9.135)
Very low level of resources			-138.568 (11.412) *	-56.813 (9.494)*	-44.187 (9.425)*	-33.053 (9.481)*
Poor			-171.387 (12.845) *	-75.971 (10.590)*	-62.003 (10.593) *	-56.028 (10.587) *
Maternal height				16.068 (0.143) *	15.908 (0.145) *	16.145 (0.144) *
Length of gestation						
22–28 weeks				-2580.491 (21.378) *	-2581.771 (21.616) *	-2566.803 (21.657) *
29–32 weeks				-1813.587 (12.011) *	-1815.726 (12.137) *	-1796117 (12.190) *
33–36 weeks				-858.069 (4.653) *	-859.875 (4.697) *	-851.399 (4.692) *
37–41 weeks (reference)				-	-	-
≥ 42 weeks				293.570 (8.623) *	294.794 (8.729) *	296.171 (8.700) *
Parity				147.626 (1.801) *	149.529 (1.818) *	150.013 (1.812) *
Maternal education						
Elementary (reference)					-43.699 (3.246) *	
Secondary (2 yrs)					-10.525 (2.448) *	
Secondary (3–4 yrs)					-	
University					-6.009 (2.929)	
Maternal smoking habits (cig/day)						
0 (reference)						-
1–9						-131.976 (3.041) *
≥ 10						-199.493 (3.970) *

***Random effects***	***Variance components***					

Intercept variance; individuals	300378.500	300183.600	300270.600	223262.800	223139.700	219758.000
(SE)	(744.144)	(744.159)	(744.340)	(587.044)	(592.469)	(586.149)
Intercept variance;neighbourhood	2498.777	2427.644	827.088	361.735	324.721	347.552
(SE)	(189.361)	(191.355)	(84.832)	(49.094)	(46.965)	(48.430)
Slope variance; year of birth	NA	11.007	9.691	8.478	7.598	8.049
(SE)		(3.970)	(3.893)	(3.282)	(3.263)	(3.289)
Covariance	NA	96.479	16.604	4.691 (9.026)	4.203 (8.796)	7.802 (8.986)
(SE)		(20.631)	(13.071)			
ICC	0.83%					
*Reduction of neighbourhood intercept variance*			66.9%	85.5%	87.0%	86.1%

NA = not applicable, ICC = intra-class correlation; SE = standard error, * = WALD-statistics > 3

In model 2 the time variable was entered as a random coefficient, allowed to vary by neighbourhood. The time trend signified an increase in ICC from 0.62 % the first year of observation to 1.7 1% the last year of observation.

In model 3, unadjusted neighbourhood-level coefficients are estimated. The reason for showing unadjusted neighbourhood estimates is that many of the individual-level controls may actually reflect the effects of prior neighbourhood conditions. Compared to the null model the between-neighbourhood variation was reduced by 66.9%. There is a linear trend in the fixed effects of neighbourhood economic status. In the poorest neighbourhoods infants on average weighed 171 g less than did infants from the most affluent neighbourhoods. The estimates are significant.

When biological factors, maternal height, length of gestation, and parity, were entered into model 4, the effect of neighbourhood economic status was only significant in the poorest neighbourhoods. The between-neighbourhood variation was reduced by an additional 19 %.

Maternal age and maternal education were highly correlated and therefore only maternal education was entered in model 5. Women with low education gave birth to smaller children. The effect was significant but when maternal smoking was included in model 6, the effect of maternal education became insignificant. The variable was therefore excluded from further analyses.

When individual-level covariates were introduced, the fixed effect of neighbourhood economic status remained significant only in the poorest neighbourhoods. In model 3 the birth weight was 172 g lower in poor neighbourhoods compared with the most affluent neighbourhoods. When significant individual-level variables were included, this effect decreased to 56 g.

## Discussion

Our results suggest that the effect on mean birth weight of neighbourhood economic status is mediated by individual gestational length and parity. This is interesting since it is line with earlier findings where woman's exposure to stress partly explains preterm birth [6,7].

Further, smoking during pregnancy is a modifier of the neighbourhood effect on birth weight. To live in a disadvantaged neighbourhood can be interpreted as a stressful exposure to the pregnant woman but can also mirror a neighbourhood where unhealthy habits like smoking during pregnancy is more socially accepted [47]. Indeed, neither gestational length nor smoking during pregnancy can be viewed as purely compositional confounders. In spite of this, we can conclude that differences in mean birth weight in Swedish urban neighbourhoods are minor.

This conclusion is supported by an intra-class correlation of less than 1%. Earlier multi-level studies in this field have not presented such components of variance, which is unfortunate. Estimates of explained between-neighbourhood variance direct focus on places instead of people and are therefore of importance to practitioners in the field of public health [48-51].

The study design is cross-sectional. However, data on exposure to a neighbourhood context preceded the outcome under study. At the time of giving birth the women had all lived in their neighbourhood for at least 1 year and most of them for a longer period of time. The validity of these exposure data can therefore be considered good. Furthermore, the study relies on register data. The Medical Birth Register has been validated and found to have good validity [37]. Also, registers linked to this register are generally considered to be of good quality [38,39].

This study is carried out in Sweden, which is described as a state with a pronounced responsibility for the welfare of the individuals, such as universal social benefits, gender equality seen in high female labour market participation, and a general and free maternal health care. We can not conclude that our negative results, i.e. the minor differences in mean birth weight in Swedish urban neighbourhoods depend on the welfare social policies in Sweden. However, Chung and Muntaner could show that more protective types of welfare regimes, namely the group of Social Democratic countries, were able to provide a more population health-friendly environment to its citizens in the last 39 years [52]. Thus, it seem reasonable to conclude that welfare institutions and benefits in Sweden might buffer against negative health outcomes such as lower mean birth weight, due to adverse structural organisation of urban neighbourhoods.

## Competing interests

The author(s) declare that they have no competing interests.

## Authors' contributions

ES outlined the design of the study, analysed the results, participated in the statistical analyses and drafted the manuscript. AH provided data and participated in the analyses of results. GA performed statistical analyses. SB participated in the design of the study and the drafting of manuscript. All authors read and approved the final manuscript.

## Pre-publication history

The pre-publication history for this paper can be accessed here:


